# Going P(u)BLIQ: Successfully Transitioning Undergraduate Medical Students from Problem-Based Learning to Inquiry Case Learning Through a Novel Hybrid Approach

**DOI:** 10.1007/s40670-024-02097-7

**Published:** 2024-06-22

**Authors:** Daniel P. Griffin, Maria Ortega, Chasity B. O’Malley

**Affiliations:** 1 Dr. Kiran C. Patel College of Allopathic Medicine, Nova Southeastern University, Fort Lauderdale, FL USA; 2 Boonshoft School of Medicine, Wright State University, Colonel Glenn Hwy, Dayton, OH USA; 3 School of Medicine, The University of Texas at Tyler, Tyler, TX USA

**Keywords:** Problem-based learning, Active learning, Inquiry case, Medical education

## Abstract

**Purpose:**

Problem-based learning (PBL) is an established learning method in medical education that uses small groups to address a “problem” in the form of a patient’s medical condition. Compared to lectures, there is compelling evidence that PBL performs better in numerous areas including student basic knowledge and satisfaction. Inquiry case (IQ) learning is a modified form of PBL, which can be described as faster-paced and requires more student responsibility. Here, a novel strategy to transition students from the PBL to IQ format is introduced that can be adopted by other programs engaging in PBL.

**Method:**

Students already engaged in PBL were introduced to the IQ format through participation in a series of hybrid PBL/IQ cases. A voluntary, anonymous survey about the hybrid system was conducted with two cohorts of students. The timing of the survey was after engaging in IQ learning following completion of the PBL/IQ hybrid system.

**Results:**

Eighty-seven of the 103 (84.47%) students completed the survey. Regarding the approach to IQ learning, students in a leader position (66.28%, *n* = 57) used journal articles as resources more than non-leaders (54.65%, *n* = 47) (*p* < 0.05). Qualitative data analysis provided insights into the biggest challenges students face as IQ learning leaders (activity creation (40%, *n* = 52/130 responses)) and non-leaders (level of preparedness (46%, *n* = 37/80 responses)). Survey responses also reported the positive impact the PBL/IQ hybrid system had on their success in IQ learning.

**Conclusions:**

This PBL/IQ hybrid method addresses the biggest challenges faced in the IQ format as leaders and non-leaders, including preparedness, time management within the sessions and in preparing for the sessions, and creating the learning experience for the second day of cases. Additionally, the hybrid approach assisted students with their transition to increased student responsibilities in the IQ format, while allowing them to develop successful strategies for success in IQ learning.

**Supplementary Information:**

The online version contains supplementary material available at 10.1007/s40670-024-02097-7.

## Introduction

Inquiry case (IQ) learning is adapted from Case Western Reserve University School of Medicine’s approach to case-based learning, now known as problem-based learning (PBL) [1]. Today, PBL is an established teaching methodology with an estimated 48% of medical schools (74 out of 153 respondents) utilizing them in the pre-clerkship curriculum, based on AAMC 2019–2020 curriculum reports of medical schools utilizing PBL for Organ System Pathophysiology [2]. The adoption of PBL in medical schools is supported by evidence of its effectiveness in student learning, development of professional skills such as self-directed learning and working collaboratively in teams, and its adaptability for integration into curricula [3]. PBL and IQ learning are exemplars of the constructivist learning theory. They present a student-centered approach that emphasizes self-directed learning, active-learning, ownership of one’s own learning, and collaboration [3, 4]. Through engaging in PBL and/or IQ learning, students are active, rather than passive, learners that are empowered to achieve understanding of content that they find important and relevant, and are provided structured opportunities for reflection on the learning process, their perception of the amount, and depth of the content learned.

Evidence of benefits of PBL over standard lecture-based delivery, as measured in health professions education, are improvements in learning outcomes such as basic knowledge [5–8], clinical skills [6, 8], collaboration skills [5], and strong evidence of student satisfaction [9].

Early studies of the impact of PBL initially provided indications that there was not a benefit in the areas of knowledge and skills, but these studies have been reviewed with scrutiny and are left with critique of how the attempts had been made to measure the impact of PBL [10]. A compelling 10-year study at University of Missouri-Columbia School of Medicine [11] compared students before and after a transition to a PBL pre-clerkship curriculum. They found that a majority of cohorts learning in a PBL pre-clerkship curriculum performed significantly better on USMLE step 1 and step 2 exams than other first-time test takers in the USA and Canada. These cohorts learning in a PBL pre-clerkship curriculum also demonstrated improved residency program director evaluations than the cohort before the transition to PBL [11].

Over the years, schools have modified PBL to suit their individual curriculum. But within its core tenants, there is rooted a fundamental structure to which institution-specific modifications have been introduced. Of these core tenants, the most notable is that at the start of the case, students are introduced to a “problem,” which in medical school is most often a patient presenting with a condition/disorder [12]. Students work in small groups that are responsible for their own learning and each group has a faculty facilitator who does not teach, and interjects only as necessary to keep the group progressing through the case. The faculty facilitator acts as support for the group, ensures the PBL session structure is followed, and provides evaluations of student individual and group performance. Students do not have knowledge of the case story beforehand, and information is meted out through sequential disclosure. A case will last multiple sessions over multiple days, typically three sessions during a week of the curriculum. The entire process involved in PBL provides important opportunities for early medical school students to practice self-directed learning and to consider how to think clinically in preparation for the clerkship phase of the curriculum and beyond their formal training.

IQ learning is a student-centered small group learning method utilized at numerous medical schools [13, 14] including Nova Southeastern University Dr. Kiran C. Patel College of Allopathic Medicine (NSU MD) [12]. IQ learning is a modified PBL that is faster paced and incorporates more student responsibilities with similar facilitator support. At NSU MD, it was decided that students would benefit from first honing their skills in PBL before beginning the more rapid and arguably more advanced IQ learning method. One example of responsibilities is that there are student roles, such as a researcher to address “quick to answer” questions, for each session of PBL and IQ learning that rotate from session to session. In PBL, each session a student is designated a “driver” and has responsibility to steer the momentum of the group to assure that the students successfully complete all of the tasks for a session. In IQ learning, the student “driver” role is replaced by the “leader” role for an entire case and this position has more critical responsibilities, as indicated below. Unlike PBL where one case is covered, commonly, over three sessions, IQ learning can introduce two complete cases in the same amount of time.

Our students engage in PBL in the first 31 weeks of the organ systems-based, pre-clerkship curriculum (Courses: Fundamentals; Hematology; Gastrointestinal, Human Nutrition, Endocrine, Reproductive (GIHNER)) and change to IQ learning for 29 weeks in the second approximately half of the pre-clerkship curriculum (Courses: Cardiovascular, Pulmonary, Renal (CPR); Brain, Body, and Behavior (BBB)). The transition from PBL to IQ learning was intentional to facilitate advanced self-directed learning within student-centered small group learning.

Initial struggles identified from the first cohort of students transitioning from PBL to IQ learning warranted a consideration of how NSU MD could support students in this process of moving from PBL to IQ learning. There was already established an *Introduction to IQ* training session in the week prior to starting IQ learning in the CPR course, but this was found to be insufficient. Students had to make significant changes to their approach to learning in the first 31 weeks of the pre-clerkship curriculum, and student IQ learning leaders had to design active learning sessions, often with little to no prior teaching experience before coming to medical school. It was imperative that the curriculum provide more of an opportunity between ending PBL and starting IQ learning sessions for students to learn to be a leader and non-leader in IQ learning.

The goal of this intervention was to introduce students to the complexities of IQ learning through a novel hybrid learning approach that utilizes skills gained through PBL to address identified student challenges with the IQ format. We hypothesized that the PBL/IQ hybrid approach would address the challenges identified by the students in leader and non-leader roles, related to the cognitive workload of an IQ format. We also hypothesized that the hybrid model would provide students with opportunities to gain confidence in their abilities to serve in these new roles of IQ learning.

## Methods

### Student Population

All first-year medical students enrolled in the Gastrointestinal, Human Nutrition, Endocrine, Reproduction (GIHNER) block participated in the hybrid format for problem-based learning (PBL).

There were 103 students in total over the two cohorts with more females (57%, *n* = 59) than males (43%, *n* = 44). The average age was 24 years and 10.7% (*n* = 11) of the cohorts had completed a master’s degree prior to enrolling in medical school. From the admission record, 1% (*n* = 1) were Native American/Pacific Islander; 19.4% (*n* = 20) were Hispanic/Latinx; 10.7% (*n* = 11) African American, Black, and/or Afro-Caribbean; 26.2% (*n* = 27) Asian; 42.7% (*n* = 44) White; 9.7% (*n* = 10) “other”; 2.9% (*n* = 3) were multiple races/ethnicity; while 7.8% (*n* = 8) did not reveal their ethnicity upon admission. Students were divided into groups of seven to eight learners per group, making 14 groups total (seven per cohort). Eighty-seven (84.47%) of the 103 students completed the survey.

### Hybrid Method of Case Delivery

Traditional PBL and IQ learning cases share a similar case design framework (see Appendix 1: General Framework for PBL/IQ Case Design), which allows for seamless creation of a hybrid format of the two styles. One popular example of traditional PBL (Table 1, column 1: Traditional PBL format) is a three-session case that runs on Monday, Wednesday, and Friday with the requirement that students continue to learn material related to the case between sessions.

**Table 1.**
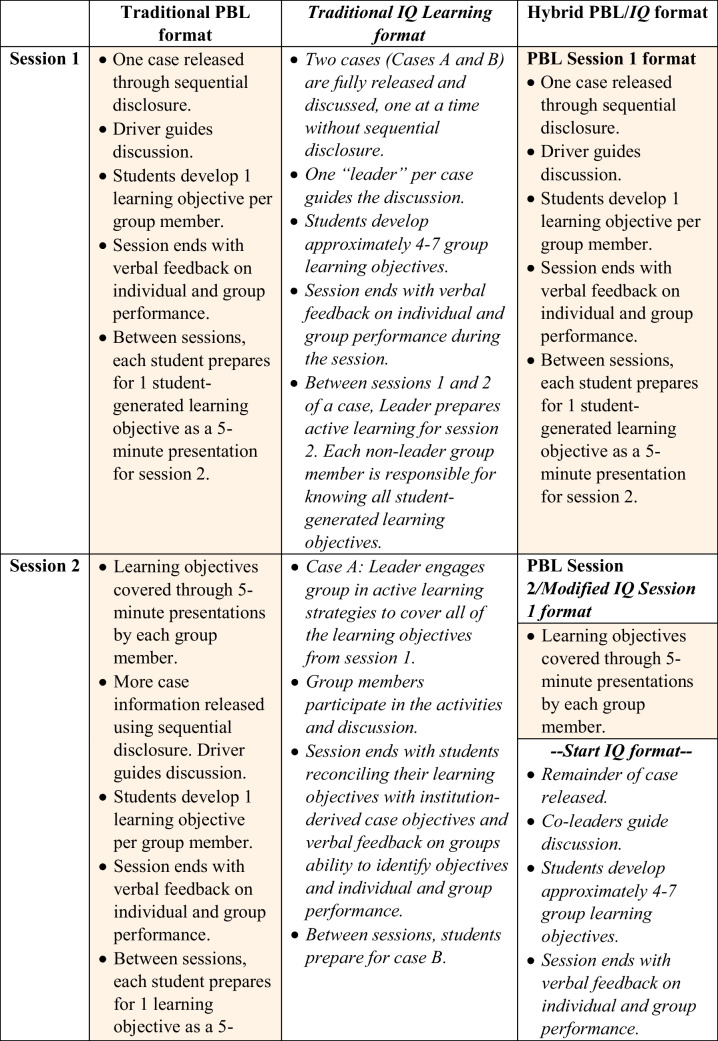

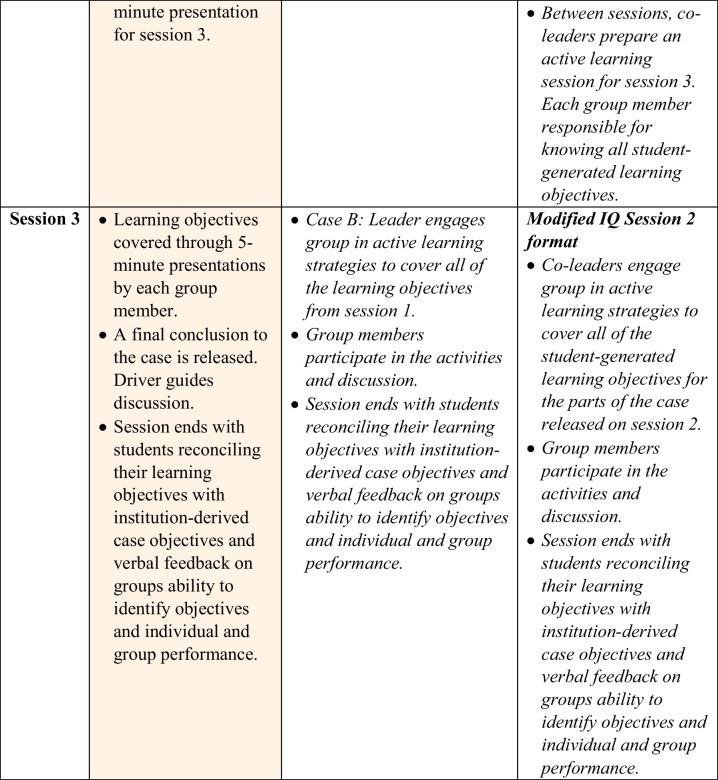
Description of problem-based learning (PBL), inquiry case (IQ) learning, and the hybrid PBL/IQ formats. Italics indicate IQ format and color filled cells with regular font indicate traditional PB

At the start of the first session (session 1) of a PBL case, students receive information through sequential disclosure of parts of a story and they must determine pertinent information to the case [10]. From this information, they form hypotheses via a broad differential diagnosis and generate questions that span from their self-identified gaps in knowledge of basic and clinical sciences, pertinent patient history, and tests they would need to run to diagnose the patient and consider treatment strategies. They continue with this process through the first session of the case. The session ends with student generation of learning objectives based on the questions they asked during the session and, finally, feedback on self- and group performance. Between session 1 and the second session (session 2), each student takes responsibility for one student-developed learning objective by researching the content using relevant and vetted resources they identify and creating a short presentation for the group. During the first approximately half of session 2, students teach the objective they were responsible for to the other members of their group. During the second approximately half of session 2, students continue the case story through sequential disclosure, and they follow the steps they took in session 1 concluding with generation of learning objectives, and group and individual feedback. Between session 2 and the third or final session (session 3), students again prepare a presentation for an objective that was created in session 2. During session 3, students begin by presenting their learning objectives in the first approximately half of the session. In the second approximately half of the session, students receive the conclusion of the case story. Institutionally derived case objectives are revealed after the case conclusion so students can reconcile these and their student-generated learning objectives with institution-derived case objectives. The final feedback at the end of session 3, where there is formal discussion of individual and group performance, includes student self-evaluation of their performance determining the most important learning objectives in comparison to the institution derived case objectives.

For IQ learning (Table 1, column 2: Traditional IQ learning format), during session 1, students receive an entire case during approximately the first half of the session, and a second case during the approximate second half of the session. Instead of sequential disclosure of the case parts, students receive the whole case and pace themselves to complete the case within the allotted time. To assist students, IQ learning cases begin with a case goal, to help student groups have a little more focus in their thinking and learning objective creation without revealing too much information about the case before beginning the story. This provides support for students in IQ learning due to the nature of the IQ learning process being more rapid than the PBL process that will cover a case in multiple sessions.

During IQ learning, students are responsible for the steps covered in session 1 of PBL for each case, as described above, but they must complete identification of pertinent information, hypotheses through more refined differential diagnosis, asking case-relevant questions, and generation of learning objectives in approximately half the time as in PBL session 1. Prior to the end of IQ learning session 1, students have completed two cases, and have student-generated learning objectives for each. The session is concluded with student and faculty facilitator feedback on individual student and group performance.

In IQ learning, instead of each student being responsible for one objective generated in a session as in PBL, they are responsible to learn all of the objectives they created before attending the second and final session of the case (session 2). In a three-session model, the second session of three of IQ learning is centered around the first case of session 1 and the third session is centered around the second case of session 1. Significant variation exists between institutions as it pertains to structure of session 2 of a case, but the intent is for students to come to the session prepared by having learned the material associated with every student-generated learning objective they developed in session 1. During session 2, they review key concepts of the case while contributing to student-centered active engagement. The end of session 2, like in session 3 of PBL, ends with students reconciling their objectives with institution-derived case objectives, and finally individual and group feedback.

At NSU MD, between sessions 1 and 2 of IQ learning, the student leader of a case learns the material as the non-leaders do, but they will also determine for each objective the most important content for each objective followed by designing active learning activities for each objective. Leaders use their creativity to create a variety of active learning activities that they feel are best to cover each learning objective, including but not limited to publicly available online polling and adapted board games. The student leader has significant responsibility in that they invest more time with the objectives, develop a plan for session 2, and create different methods to engage the group to have them apply their learning of the student-generated learning objectives.

The PBL/IQ hybrid model (See Table 1, column 3: Hybrid PBL/IQ format) was designed to allow a significant amount of case information to be introduced in the PBL format for which students were well trained after having engaged in it for almost half of the pre-clerkship curriculum. In this format, students engage in one case over three sessions as they would in PBL, maintaining the number of cases and intended content, but providing a new format. The standard PBL format is used session 1 and the first approximately half of session 2. In the second approximately half of session 2, students receive the rest of the case, including the conclusion normally provided during PBL session 3. This is designed for students to manage the timing and pacing of the case the same session time constraints as they will have in IQ learning. Session 2 concludes with developing student-generated learning objectives and feedback. Session 3 of the PBL/IQ hybrid case is delivered similar to session 2 of a standard IQ learning case.

Before a student needs to independently act as a case leader in IQ learning, fulfilling the great responsibilities of a leader, the PBL/IQ hybrid teams two students as co-leaders so that they can better manage the time commitment and support each other in designing their active learning session. The feedback at the end of each PBL/IQ hybrid session contains focused questions (see questions provided as Supplemental Digital Appendix 2) that address student experiences as leaders and non-leaders including challenges and successful strategies they used, in the IQ format, so they can support and learn from each member of the group and receive advice from the faculty facilitator.

NSU MD employs four cases for the PBL/IQ hybrid format. This is based on group size to ensure that every student in a group has at least one chance to act as co-leader of a case before moving to standard IQ learning in the next course. This direct experience as a co-leader is complemented with student discussions at the end of each case where they reflect on their experiences and support each other with feedback and suggestions to address difficulties they had in the roles. Between the GIHNER and CPR course, and between PBL and IQ learning, a session is held with the students in a large group setting to provide review of the IQ format and active engagement with the whole class so that every group has a chance to discuss with other groups their experiences, and strategies they found to be successful in engaging in IQ learning.

### Project Timeline

Students formed new PBL groups of seven to eight members at the start of the GIHNER course and then participated in the hybridized PBL/IQ format for the final four of nine PBL cases. This allowed them to form a cohesive PBL group and then having four cases allowed all students to act as co-leader at least once. The final four cases followed the traditional 3 days per week format, but were modified to fit the novel hybrid approach.

During the subsequent course (Cardiovascular, Pulmonary, and Renal, known as CPR), students participated in a full IQ format for 19 cases. During the final week of the CPR course, students participated in the voluntary, anonymous survey to assess the perceptions of effectiveness of the PBL/IQ hybrid. Students were informed in advance that the voluntary survey would be given at the end of a CPR session to those who opted to participate.

### Survey Instrument

The survey consisted of 19 questions, including a 5-point Likert scale, yes/no, and free response questions (full survey available as Supplemental Digital Appendix 3). Survey items assessed attitudes and preparedness of students for the IQ learning sessions, along with narrative free response for direct feedback on the hybrid model. Likert scale consisted of the following: 1 = strongly disagree, 2 = disagree, 3 = neither agree nor disagree, 4 = agree, 5 = strongly agree. It was distributed through RedCap using electronic consent.

### Data Analysis

We used IBM SPSS 28.0 statistics package for data analysis. Descriptive statistics were used for demographic data. Pearson correlations and paired *t*-tests, with *p* < 0.05 were considered significant. We calculated Cronbach’s alpha to determine internal reliability of the survey questions.

This study was designated as exempt from the Nova Southeastern University Institutional Review Board (Protocol 2021–276-NSU).

## Results

**Fig. 1:**
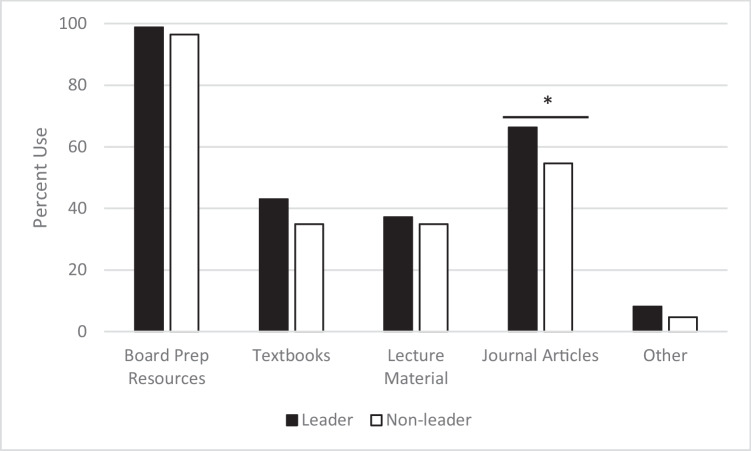
Preparation differences as leader vs non-leader for the PBL/IQ hybrid format. Self-reported usage (in percent) of preparation materials by 87 first year medical students who engaged in the PBL/IQ hybrid format from 2020 to 2021, *p < 0.05

There was a difference in the resources used for preparation students used for the activities as a leader verses a non-leader. Use of board prep resources (98.83% (*n* = 85) as leader vs 96.51% (*n* = 83) as non-leader), textbooks (43.02% (*n* = 37) as leader vs 34.88% (*n* = 30) as non-leader), lecture materials (37.21% (*n* = 32) as leader vs 34.88% (*n* = 30) as non-leader), and other resources (8.13% (*n* = 7) as leader vs 4.65% (*n* = 4) as non-leader) were similar between the two roles (Fig. 1). There was a significant increase in the use of journal articles for preparation in the leaders (66.28%, *n* = 57) compared to the non-leaders (54.65%, *n* = 47) (*p* < 0.05) (Fig. 1).

**Fig. 2:**
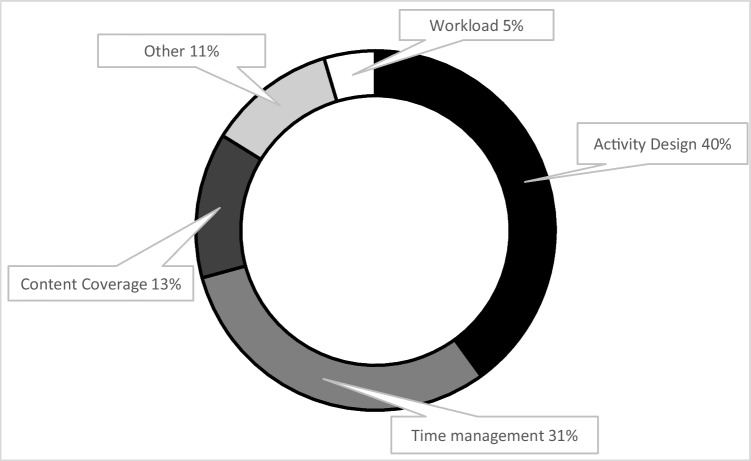
Challenges as a leader in PBL to IQ learning transition. Qualitative theme analysis of the 130 open-ended question responses from students regarding the leader role

**Fig. 3:**
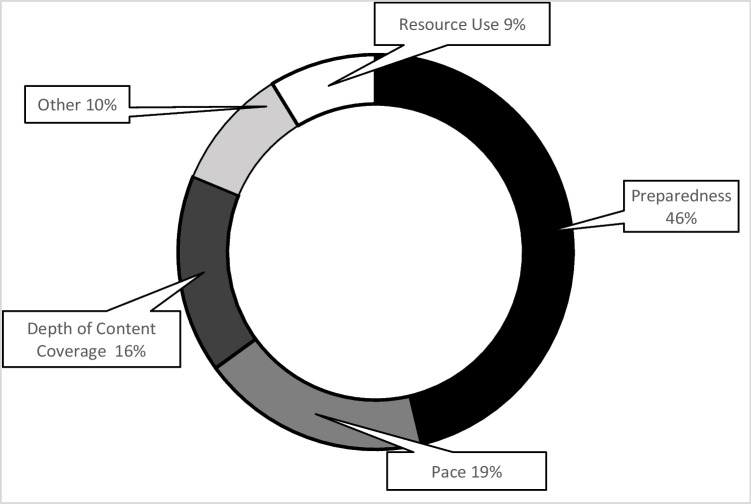
Challenges as a non-leader in PBL to IQ learning transition. Qualitative theme analysis of the 80 open-ended question responses from students regarding the non-leader role

Using qualitative theme analysis for the open-ended questions, students showed there were differences in the perceived challenges of IQ learning in leader roles compared to non-leader roles. Leaders found the biggest challenges to be designing the activities (40%, *n* = 52/130 responses), time management (31%, *n* = 40/130 responses), content coverage (13%, 17/130 responses), other (11%, *n* = 15/130 responses), and workload (5%, *n* = 6/130 responses) (Fig. 2). Non-leaders found the biggest challenges to be level of preparedness (46%, *n* = 37/80 responses), pace (19%, *n* = 15/80 responses), depth of content coverage (16%, *n* = 13/80 responses), other (10%, *n* = 8/80 responses), and resource use (9%,* n* = 7/80 responses) (Fig. 3). Other responses as leader included detailed case presentation, knowing the expectations, amount of work, being the leader, facilitating group discussion, knowing when to serve as leader, lack of guidance, unclear information, remembering format, and more focus on clinical reasoning. As non-leader, other responses included understanding requirements, activities that required writing practice problems, determining how to contribute to the sessions, preparing the study guide, and coming up with objectives.

**Fig. 4:**
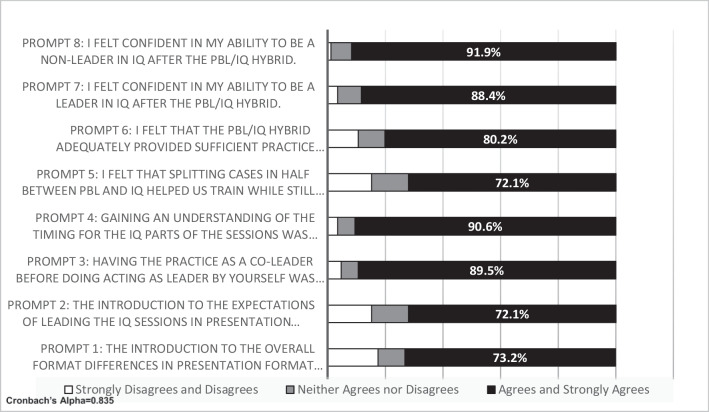
Survey responses to Likert-scale prompt questions related to the PBL/IQ hybrid approach. Likert scale consisted of the following: 1 = strongly disagree, 2 = disagree, 3 = neither agree nor disagree, 4 = agree, 5 = strongly agree. Responses were grouped as strongly disagree and disagree, neither agree nor disagree, and agree and strongly agree. Cronbach’s alpha was 0.835, *n* = 87

Overall, survey responses to the PBL/IQ hybrid were favorable. Ninety-two percent of the participants (*n* = 80) agreed or strongly agreed that they felt confident in their abilities to be a non-leader in IQ learning after the PBL/IQ hybrid. Eighty-eight percent of participants agreed or strongly agreed that they felt confident in their ability to be a leader after the hybrid experience (*n* = 77) (Fig. 4). Eighty percent of participants agreed or strongly agreed that the hybrid provided sufficient practice preparing for IQ learning (*n* = 70). Seventy-two percent agreed or strongly agreed that splitting the cases helped them train and that the introduction for the expectations of hybrid was helpful (*n* = 63). Additionally, 89.5% of the participants agreed or strongly agreed that having practice as co-leader was helpful for the IQ learning transition (*n* = 78). Additionally, 91% of the participants agreed or strongly agreed that they felt it was helpful to gain an understanding of the timing for IQ learning during the hybrid (*n* = 79) (Fig. 4). Seventy-three percent of participants agreed or strongly agreed that the introductory session for the format of the hybrid was helpful for the transition to IQ learning (*n* = 64). A Cronbach’s alpha for the survey was calculated at 0.835 indicating a high reliability for the survey responses (Fig. 4).

There was a moderate positive correlation between participants who showed increases in student confidence with their ability to be a leader in IQ learning after the PBL/IQ hybrid (prompt 7) with agreeing that having practice as co-leader before doing acting as leader by yourself was helpful in preparing for IQ learning (prompt 3), *r* = 0.489, *N* = 86, *p* < 0.001 (Available as Supplemental Digital Appendix 4).

There was a moderate positive correlation between participants who showed an increase in student confidence with their ability to be a leader in IQ learning after the PBL/IQ hybrid (prompt 7) with agreeing that the PBL/IQ hybrid adequately provided sufficient practice preparing (e.g., time management) for all objectives in IQ learning instead of a single objective in PBL (prompt 6) (*r* = 0.538, *N* = 86, *p* < 0.001) (Available as Supplemental Digital Appendix 4). There was a moderate positive correlation between participants who showed an increase in student confidence with their ability to be a leader in IQ learning after the PBL/IQ hybrid (prompt 7) with also feeling confident in their ability to be a non-leader after the PBL/IQ hybrid (prompt 8) (*r* = 0.610, *N* = 86, *p* < 0.001) (Available as Supplemental Digital Appendix 4).

There was a moderate positive correlation between participants who felt that having the practice as a co-leader before acting as leader by themselves was helpful in preparing for IQ learning (prompt 3) with also having increased feelings that the PBL/IQ hybrid adequately provided sufficient practice preparing (e.g., time management) for all objectives in IQ learning instead of a single objective in PBL (prompt 6) (*r* = 0.482, *N* = 86, *p* < 0.001) (Available as Supplemental Digital Appendix 4).

There was a moderate positive correlation between participants who felt that having the practice as a co-leader before acting as leader by themselves was helpful in preparing for IQ learning (prompt 3) with demonstrating an increase in confidence in their ability to be a non-leader in IQ learning after the PBL/IQ hybrid (prompt 8) (*r* = 0.444, *N* = 86, *p* < 0.001) (Available as Supplemental Digital Appendix 4).

## Discussion

The switch from PBL to IQ learning for students was designed as a shift in learning to maintain student engagement with small group learning and to expand on their developing skills for critical thinking. Students first learned how to perform the necessary steps of PBL at a slower pace early in their learning and development of clinical decision-making, students are ready to approach clinical problems, incorporating basic science knowledge, more quickly and in a way that simulates the considerations and decision-making they will engage in clinical simulation and clinical settings. IQ learning also requires more student ownership of the sessions providing a more student self-directed learning experience. From a student’s perspective “I loved the opportunity to teach the group which forced me to truly immerse in the material and hone my skills to be the best expert I could be as a leader. I also appreciate the opportunity to think and execute creative activities that many of my group members enjoyed.” In terms of the co-leader aspect, one student reported that they liked “being able to get some experience as a leader without having the full weight of preparing a session fall on me alone.”

The format change from PBL to IQ learning can be jarring to students for several reasons including  the increased case load (from one to two cases per week); new, high-stakes responsibilities of a student to lead an IQ learning session independently; and the requirement that each student learn numerous objectives to the same level of mastery as they were previously expected to learn for one objective in PBL.

This hybrid model was designed with the intent of providing students with sufficient time to learn what they need to do to be successful as an IQ learning team member and team leader with more support than they would otherwise have if they moved directly from PBL to IQ formats. One student reported “I enjoy the aspect of becoming as much of an expert on a specific topic as possible. I did gain quite a bit in teaching others and also learning from them during each session. The PBL/IQ hybrid training offered a low stakes way to make the transition from PBL to IQ learning, without it the transition would have been much more difficult,” while another reported “it [the hybrid] lessened the stress of the IQ [learning] transition.”

We placed the PBL/IQ hybrid approximately halfway through the pre-clerkship curriculum, following two iterations of traditional PBL and halfway through the GIHNER course to allow for efficient new group formation during the first half of the course. Through the first five cases in the course, students engage in the Forming, Storming, Norming, and Performing [15] of team formation with assistance as necessary from the faculty facilitator prior to transitioning to the PBL/IQ hybrid for the remaining/last four cases. Students reported liking that the hybrid “allowed adjustment period and clarifying questions relating to the transition to IQ [learning].”

### Preparation

Students indicated a considerable reliance on board preparation materials (e.g., USMLE STEP 1 study resources) in developing and preparing for the second day of IQ learning sessions, with less utilization of textbooks and lecture materials. One factor that may contribute to the lower usage of lecture materials is that at NSU MD, PBL and IQ learning is integrated into the curriculum, but is often a source of new material that is not covered in other sessions, such as lectures. One unanticipated and welcome finding from this PBL/IQ hybrid initiative was a greater usage of current literature by team leaders. When students are in control of developing learning activities, they are not relying solely on board-preparation materials, but deciding to give importance to current advancements in the scientific and clinical fields as they continue to develop their skills as lifelong learners. This also contributes to the goals of students engaging in accreditation requirements of self-directed and lifelong learning as outlined in Element 6.3 of the LCME Standards [16] as they develop these invaluable skills they will use as practicing clinicians.

### Challenges

As anticipated, and as a catalyst for inception of this PBL/IQ hybrid strategy, survey results showed that there was a challenge shared by the leaders and non-leaders of preparation time for the IQ learning sessions. A benefit of students partaking in IQ learning is that students learn and strive to master material for all of the student-generated learning objectives for each case in session 2, instead of one objective as in PBL, which helps distribute the student learning over the weeks before their formal assessments. With this, students need to adapt their previous learning strategies to allow for sufficient time to prepare for IQ learning. Despite the preparation time being a challenge, one student noted that “The change of pace from PBL to a more individualized IQ [learning]” when asked what they enjoyed the most about the PBL/IQ hybrid. In the hybrid, each PBL/IQ case has a PBL style of preparation and IQ learning style of preparation, which eases students into learning how to incorporate more time learning toward case objectives without having all of the learning for the case reliant on successfully adopting a new schedule. Of respondents, 72.1% agreed or strongly agreed that splitting the case between PBL and IQ formats helped them train in the IQ learning process while still learning the necessary material.

### Activities Creation by Leader

Of great import is that the IQ format relies on students being able to, as leaders, independently develop a session to engage their teammates in learning. Students are not expected to understand curricular design when they enter medical school, and very few have teaching backgrounds, which creates significant pressure on a student leader to develop a learning session. In the PBL/IQ hybrid model, this is addressed by having two co-leaders work together in developing and implementing their learning session. This first reduces the amount of time it takes to develop a session compared to an independent leader. It also provides a team approach to identifying, creating, and implementing the best methods to engage the group in their learning. Students appreciated the opportunity to be a co-leader, stating “I liked being a co-leader. I agree that it would have been too difficult for me to handle all by myself on the first try.” Students also reported that the experience they gained as co-leaders in the PBL/IQ format was helpful in preparation for individually leading the group (89.5% of respondents agreed to strongly agreed) and they were confident in their abilities to be IQ learning leaders after they engaged in the PBL/IQ hybrid (88.4% of respondents agreed to strongly agreed). The goals of the PBL/IQ hybrid approach to prepare students for IQ learning as leaders and team members were successful.

## Future Directions

Future directions for this hybrid system include addressing the student’s self-identified challenge of knowing the depth of content knowledge necessary as leaders and non-leaders, with 13% and 16% of respondents identifying this as a biggest challenge in IQ learning, respectively. Informal feedback from course directors and faculty facilitators, as well as student survey responses, support that creating learning objectives with enough depth of content coverage is an area in need of attention. Realizing content depth is not unique to the PBL/IQ hybrid, but this format can be a component in training of students in a more longitudinal fashion across the curriculum to ensure that they are gaining the skills necessary to become proficient in self-directed and lifelong learning.

## Conclusion

This PBL/IQ hybrid approach was successful in addressing the student-identified biggest challenges faced in the IQ format as IQ learning leaders and non-leaders, including preparedness, time management within the sessions and in preparing for the sessions, and creating the learning experience for the second day of the cases. With students paired as co-leaders, they were able to practice the role of leader while sharing the responsibilities which reduced their effort. Taken together, this hybrid delivery has resulted in a favorable approach of engaging students in their transition to the more intense self-directed, case-based learning method of IQ learning. This approach could be adapted to other programs interested in using both PBL and IQ learning as case-based learning methods.

## Supplementary Information

Below is the link to the electronic supplementary material.

Supplementary file1 (PDF 134 KB)

Supplementary file2 (PDF 65 KB)

Supplementary file3 (PDF 94 KB)

Supplementary file4 (PDF 138 KB)

## Data Availability

The datasets generated during and/or analyzed during the current study are available from the corresponding author on reasonable request.
