# Supplementary material for: Going P(u)BLIQ: Successfully Transitioning Undergraduate Medical Students from Problem-Based Learning to Inquiry Case Learning Through a Novel Hybrid Approach
Source: Med Sci Educ. 2024 Jun 22;34(5):1079–89. doi: 10.1007/s40670-024-02097-7 (PMC11496440; doi:10.1007/s40670-024-02097-7)
Supplement: Supplementary file 1 — Supplementary file1 (PDF 134 KB) [file 40670_2024_2097_MOESM1_ESM.pdf]

Going P(u)BLIQ: Successfully transitioning undergraduate medical students from Problem Based Learning to Case Inquiry learning through a novel hybrid approach

Medical Science Educator

Authors: Daniel P. Griffin, PhD[1, 3]; Maria Ortega, MPA[1]; Chasity B. O'Malley, PhD[1, 2]

Affiliations: [1]Dr. Kiran C. Patel College of Allopathic Medicine, Nova Southeastern University, Fort Lauderdale, FL
[2]Boonshoft School of Medicine, Wright State University, Dayton, OH
[3]University of Texas at Tyler School of Medicine, Tyler, TX

Correspondence should be addressed to Chasity B. O'Malley; chasity.omalley@wright.edu; 3640 Colonel Glenn Hwy., Dayton, Ohio 45435

General Framework for PBL/IQ Case Design:

Patient presents with symptoms; students develop differential diagnoses and based on the given information, develop relevant questions about what they would like to learn/know before continuing with the case

Patient's history of present illness is given; students refine differential diagnoses and questions they want to know

Patient's past medical history is given; students refine differential diagnoses and questions they want to know and determine how to approach the physical exam

Patient's physical exam is given/findings described; students refine differential diagnoses and questions they want to know and students consider what diagnostics need to be done

Patient has initial diagnostic tests done/results are given; students refine differential diagnoses and questions they want to know and students determine if additional diagnostics are needed

Students determine the diagnosis of the patient/diagnosis revealed (not all cases contain a diagnosis); students determine appropriate treatment for the patient

Follow up testing is identified/results shared where appropriate
