# Supplementary material for: Going P(u)BLIQ: Successfully Transitioning Undergraduate Medical Students from Problem-Based Learning to Inquiry Case Learning Through a Novel Hybrid Approach
Source: Med Sci Educ. 2024 Jun 22;34(5):1079–89. doi: 10.1007/s40670-024-02097-7 (PMC11496440; doi:10.1007/s40670-024-02097-7)
Supplement: Supplementary file 2 — Supplementary file2 (PDF 65 KB) [file 40670_2024_2097_MOESM2_ESM.pdf]

**Going P(u)BLIQ: Successfully transitioning undergraduate medical students from Problem Based Learning to Case Inquiry learning through a novel hybrid approach**

**Medical Science Educator**
**Authors:** Daniel P. Griffin, PhD[1, 3]; Maria Ortega, MPA[1]; Chasity B. O'Malley, PhD[1, 2]

**Affiliations:** [1]Dr. Kiran C. Patel College of Allopathic Medicine, Nova Southeastern University, Fort Lauderdale, FL
[2]Boonshoft School of Medicine, Wright State University, Dayton, OH
[3]University of Texas at Tyler School of Medicine, Tyler, TX

**Correspondence should be addressed to** Chasity B. O'Malley; chasity.omalley@wright.edu; 3640 Colonel Glenn Hwy., Dayton, Ohio 45435 ORCID 0000-0002-5362-0946

**Supplemental Digital Appendix 2: Post-Session Feedback Questions for PBL/IQ Learning Hybrid**

1. What were the biggest challenges transitioning from PBL to IQ format (leader and non-leader roles)?

2. What effective strategies did you use to prepare for your IQ leadership responsibilities?

3. What effective strategies did you use to prepare to engage in IQ as a member of the team (non-leader role)?

4. What would be most helpful to your training in IQ that could be introduced during these PBL/IQ hybrid cases?

5. What did you enjoy the most about this transition to IQ using the PBL/IQ hybrid case?
