# Supplementary material for: Going P(u)BLIQ: Successfully Transitioning Undergraduate Medical Students from Problem-Based Learning to Inquiry Case Learning Through a Novel Hybrid Approach
Source: Med Sci Educ. 2024 Jun 22;34(5):1079–89. doi: 10.1007/s40670-024-02097-7 (PMC11496440; doi:10.1007/s40670-024-02097-7)
Supplement: Supplementary file 3 — Supplementary file3 (PDF 94 KB) [file 40670_2024_2097_MOESM3_ESM.pdf]

Going P(u)BLIQ: Successfully transitioning undergraduate medical students from Problem Based Learning to Case Inquiry learning through a novel hybrid approach

Medical Science Educator

Authors: Daniel P. Griffin, PhD[1, 3]; Maria Ortega, MPA[1]; Chasity B. O'Malley, PhD[1, 2]

Affiliations: [1]Dr. Kiran C. Patel College of Allopathic Medicine, Nova Southeastern University, Fort Lauderdale, FL
[2]Boonshoft School of Medicine, Wright State University, Dayton, OH
[3]University of Texas at Tyler School of Medicine, Tyler, TX

Correspondence should be addressed to Chasity B. O'Malley; chasity.omalley@wright.edu; 3640 Colonel Glenn Hwy., Dayton, Ohio 45435 ORCID 0000-0002-5362-0946

Supplemental Digital Appendix 3: Student Survey Instrument

The goal of this survey is to assess students' attitudes regarding the transition from Problem Based Learning (PBL) to Inquiry Case Learning (IQ). In particular, we are interested in evaluating how the hybrid of PBL/IQ experienced in the Gastrointestinal/Human Nutrition/Endocrine/Reproduction (GIHNER) block aided in the transition into the IQ format for the Cardiovascular/Pulmonary/Renal (CPR) block. Now that you have completed IQ in the CPR block, we ask you to reflect on the PBL/IQ hybrid delivery in GIHNER. Please remember that participation in this survey is completely anonymous and voluntary. We appreciate your feedback!

Please answer the following questions as completely as possible, providing details on the free response questions.

(Likert) From the different aspects of the PBL/IQ hybrid, please rate the following on a scale of 1-5 to indicate which was most useful in IQ for CPR?

Please note that a 1 indicates strongly disagrees, 2 disagrees, 3 neither agrees nor disagrees, 4 agrees, and 5 strongly agrees. If none of the answers are applicable, please use the N/A. The introduction to the overall format differences in presentation format was helpful in preparing for IQ

1. The introduction to the expectations of leading the IQ sessions in presentation format was helpful in preparing for IQ
2. Having the practice as a co-leader before doing acting as leader by yourself was helpful in preparing for IQ
3. Gaining an understanding of the timing for the IQ parts of the sessions was helpful in preparing for IQ
4. I felt that splitting cases in half between PBL and IQ helped us train while still learning the material necessary in the case
5. I felt confident in my ability to be a leader in IQ after the PBL/IQ hybrid.

6. I felt confident in my ability to be a non-leader in IQ after the PBL/IQ hybrid.

(Narrative) As a leader, what was the biggest challenge transitioning from PBL to IQ?

(MCQ) Do you feel that the PBL/IQ hybrid from GIHNER helped you prepare for this challenge?

A. Yes
B. No

(MCQ) Do you feel that being an IQ co-leader in GIHNER was helpful before being the only leader in CPR?

A. Yes
B. No

(Narrative) As a non-leader, what was the biggest challenge transitioning from PBL to IQ?

(MCQ) Do you feel that the PBL/IQ hybrid from GIHNER helped you prepare for this challenge?

A. Yes
B. No

(MCQ) How did you prepare for IQ leadership? Please check all that apply.

A. Reviewed board prep resources (Amboss, First Aid, Boards and Beyond, Sketchy, etc…)
B. Reviewed the textbooks
C. Reviewed lecture material
D. Consulted journal articles
E. Something not listed

(Narrative) If you indicated something not listed in the question above, please elaborate here.

(MCQ) How did you prepare for IQ sessions as a non-leader? Please check all that apply.

A. Reviewed board prep resources (Amboss, First Aid, Boards and Beyond, Sketchy, etc…)
B. Reviewed the textbooks
C. Reviewed lecture material
D. Consulted journal articles
E. Something not listed

(Narrative) If you indicated something not listed in the question above, please elaborate here.

(MCQ) Was your preparation for an IQ session different from your preparation for PBL?

A. Yes
B. Yes, but only as a leader
C. Yes, but only as a non-leader

D. No

(Narrative) What would you have liked to see in the PBL/IQ hybrid training that would have helped for the IQ transition?

(Narrative) What changes would you like to see to the PBL/IQ hybrid training, if any?

(Narrative) What did you enjoy the most in the IQ/PBL hybrid?
