# Supplementary material for: Going P(u)BLIQ: Successfully Transitioning Undergraduate Medical Students from Problem-Based Learning to Inquiry Case Learning Through a Novel Hybrid Approach
Source: Med Sci Educ. 2024 Jun 22;34(5):1079–89. doi: 10.1007/s40670-024-02097-7 (PMC11496440; doi:10.1007/s40670-024-02097-7)
Supplement: Supplementary file 4 — Supplementary file4 (PDF 138 KB) [file 40670_2024_2097_MOESM4_ESM.pdf]

**Going P(u)BLIQ: Successfully transitioning undergraduate medical students from Problem Based Learning to Case Inquiry learning through a novel hybrid approach**

**Medical Science Educator**
**Authors:** Daniel P. Griffin, PhD[1, 3]; Maria Ortega, MPA[1]; Chasity B. O'Malley, PhD[1, 2]

**Affiliations:** [1]Dr. Kiran C. Patel College of Allopathic Medicine, Nova Southeastern University, Fort Lauderdale, FL
[2]Boonshoft School of Medicine, Wright State University, Dayton, OH
[3]University of Texas at Tyler School of Medicine, Tyler, TX

**Correspondence should be addressed to** Chasity B. O'Malley; chasity.omalley@wright.edu; 3640 Colonel Glenn Hwy., Dayton, Ohio 45435 ORCID 0000-0002-5362-0946

**Supplemental Digital Appendix 4: Pearson Correlation Table**

| | | Prompt 1 | Prompt 2 | Prompt 3 | Prompt 4 | Prompt 5 | Prompt 6 | Prompt 7 | Prompt 8 |
|---|---|---|---|---|---|---|---|---|---|
| Prompt 1 | Pearson Correlation | 1 | .725** | .291** | .505** | .314** | .321** | .231* | 0.134 |
| | Sig. (2-tailed) | | 0.000 | 0.007 | 0.000 | 0.003 | 0.003 | 0.032 | 0.219 |
| | N | 86 | 86 | 86 | 85 | 86 | 86 | 86 | 86 |
| Prompt 2 | Pearson Correlation | .725** | 1 | .254* | .387** | .360** | .306** | .280** | .236* |
| | Sig. (2-tailed) | 0.000 | | 0.018 | 0.000 | 0.001 | 0.004 | 0.009 | 0.029 |
| | N | 86 | 86 | 86 | 85 | 86 | 86 | 86 | 86 |
| Prompt 3 | Pearson Correlation | .291** | .254* | 1 | .287** | .426** | .482** | .489** | .444** |
| | Sig. (2-tailed) | 0.007 | 0.018 | | 0.008 | 0.000 | 0.000 | 0.000 | 0.000 |
| | N | 86 | 86 | 86 | 85 | 86 | 86 | 86 | 86 |
| Prompt 4 | Pearson Correlation | .505** | .387** | .287** | 1 | .429** | .390** | .309** | .392** |
| | Sig. (2-tailed) | 0.000 | 0.000 | 0.008 | | 0.000 | 0.000 | 0.004 | 0.000 |
| | N | 85 | 85 | 85 | 85 | 85 | 85 | 85 | 85 |
| Prompt 5 | Pearson Correlation | .314** | .360** | .426** | .429** | 1 | .702** | .371** | .424** |
| | Sig. (2-tailed) | 0.003 | 0.001 | 0.000 | 0.000 | | 0.000 | 0.000 | 0.000 |
| | N | 86 | 86 | 86 | 85 | 86 | 86 | 86 | 86 |
| Prompt 6 | Pearson Correlation | .321** | .306** | .482** | .390** | .702** | 1 | .538** | .519** |
| | Sig. (2-tailed) | 0.003 | 0.004 | 0.000 | 0.000 | 0.000 | | 0.000 | 0.000 |
| | N | 86 | 86 | 86 | 85 | 86 | 86 | 86 | 86 |
| Prompt 7 | Pearson Correlation | .231* | .280** | .489** | .309** | .371** | .538** | 1 | .610** |
| | Sig. (2-tailed) | 0.032 | 0.009 | 0.000 | 0.004 | 0.000 | 0.000 | | 0.000 |
| | N | 86 | 86 | 86 | 85 | 86 | 86 | 86 | 86 |
| | Pearson Correlation | 0.134 | .236* | .444** | .392** | .424** | .519** | .610** | 1 |
| | Sig. (2-tailed) | 0.219 | 0.029 | 0.000 | 0.000 | 0.000 | 0.000 | 0.000 | |
| | N | 86 | 86 | 86 | 85 | 86 | 86 | 86 | 86 |
| **. Correlation is significant at the 0.01 level (2-tailed). | | | | | | | | | |
| *. Correlation is significant at the 0.05 level (2-tailed). | | | | | | | | | |

Prompt 1: The introduction to the overall format differences in presentation format was helpful in preparing for IQ.

Prompt 2: The introduction to the expectations of leading the IQ sessions in presentation format was helpful in preparing for IQ.

Prompt 3: Having the practice as a co-leader before doing acting as leader by yourself was helpful in preparing for IQ.

Prompt 4: Gaining an understanding of the timing for the IQ parts of the sessions was helpful in preparing for IQ.

Prompt 5: I felt that splitting cases in half between PBL and IQ helped us train while still learning the material necessary in the case.

Prompt 6: I felt that the PBL/IQ hybrid adequately provided sufficient practice preparing (e.g. time management) for all objectives in IQ instead of a single objective in PBL.

Prompt 7: I felt confident in my ability to be a leader in IQ after the PBL/IQ hybrid.

Prompt 8: I felt confident in my ability to be a non-leader in IQ after the PBL/IQ hybrid.
